# The Treatment of Seborrhœa of the Scalp

**Published:** 1899-11-11

**Authors:** 


### THE TREATMENT OF SEBORRHŒA OF THE SCALP.

In the course of a clinical demonstration at the West London Hospital on a case of alopecia areata, Dr. Phineas Abraham[1] said that we must look upon the baldness of alopecia areata as possibly caused in several ways. Occasionally it is a sequel of ringworm, but more often it follows seborrhœa, or an eczema of the sebaceous glands and hair follicles. Sabouraud found the same bacillus in the mouths of the follicles which Unna found in the seborrhœic eczema. The seborrhœic condition of the scalp can be cured in several ways. The favourite way of Dr. Unna is to apply some preparation of sulphur. Resorcin is also good, or sulphur and resorcin mixed may be used. At the Blackfriars Hospital for Skin Diseases the method used is the red oxide of mercury 10 to 15 grains to the ounce. At the West London Hospital there is a complicated formula containing red oxide and red sulphide of mercury and creasote, which cures these cases perfectly well. Alopecia also sometimes follows impetigo, and in other cases it appears after fevers.

[1] Clinical Journal, Oct. 18.

